# Genome-wide identification of WRKY gene family members in *Populus trichocarpa* and their response to biotic stresses

**DOI:** 10.7717/peerj.21132

**Published:** 2026-04-13

**Authors:** Yue Sun, Hanxi Li, Yao Chi, Yanlin Liu, Xinxin Zhang, Xiyang Zhao

**Affiliations:** 1 Jilin Provincial Key Laboratory of Tree and Grass Genetics and Breeding, College of Forestry and Grassland, Jilin Agriculture University, Changchun, China; 2 Tree Genetics and Breeding, Northeast Forestry University, Harbin, China

**Keywords:** Populus trichocarpa, WRKY gene family, Genome-wide identification, Hyphantria cunea

## Abstract

**Background:**

*WRKY* transcription factors (TFs) are key regulators of plant stress resistance. Despite their importance, the regulatory mechanisms of *WRKY*s in *Populus trichocarpa* under biotic stress remain insufficiently explored. This study presents a comprehensive bioinformatic characterization of the *WRKY* gene family in *P. trichocarpa*.

**Methods:**

The most recent *P. trichocarpa* genome was employed to identify all *WRKY* genes in the species. Detailed analyses, including molecular characterization, phylogenetic relationships, gene structure, motif and promoter composition, chromosomal localization, and syntenic relationships, were conducted. Real-time quantitative polymerase chain reaction (RT-qPCR) was utilized to assess the expression profiles of *PtWRKY* genes under insect stress.

**Results:**

A total of 139 *WRKY* genes were identified, unevenly distributed across 18 chromosomes and classified into three principal groups. Conserved motif analysis showed that all *PtWRKY* proteins contained motifs 1, 2, and 4, with members of the same subgroup exhibiting highly similar motif architectures. Collinearity analysis identified 91 homologous gene pairs within the *PtWRKY* family, suggesting potential functional redundancy. Examination of the 2,000 bp upstream promoter regions revealed diverse cis-acting elements associated with light responsiveness, phytohormone signaling, and stress regulation. Moreover, several *PtWRKY* genes responded to feeding stress caused by *Hyphantria cunea*.

**Conclusion:**

Collectively, these findings elucidate the genomic organization and potential regulatory roles of the WRKY gene family in *P. trichocarpa*, highlighting their involvement in insect-induced defense and offering insights for future agricultural improvement strategies.

## Introduction

Herbivorous insects are significant pests in agriculture and forestry, causing substantial economic and ecological losses. In response to herbivory, plants activate signaling pathways that trigger specific defense mechanisms critical for their survival. Extensive research has established transcription factors (TFs) as key regulators of gene expression, playing pivotal roles in plant defense against herbivores (Romani & Moreno, 2021; Jiang et al., 2017; Phukan, Jeena & Shukla, 2016). By binding to cis-regulatory elements in the promoter regions of target genes, TFs modulate gene expression and facilitate coordinated crosstalk among signal transduction pathways (Javed & Gao, 2023; Priest, Filichkin & Mockler, 2009; Shrestha, Khan & Dey, 2018), ultimately strengthening plant resistance to stress. Given their role as master regulators of defense-related genes, TFs present promising candidates for genetic engineering applications (Chen et al., 2017). Consequently, understanding the mechanistic actions of TFs is vital for advancing insect resistance research.

The *WRKY* TF was first identified in sweet potato (*Ipomoea batatas*) (Ishiguro & Nakamura, 1994). Subsequent systematic analysis of the *WRKY* gene family have been conducted in various plants, including Arabidopsis, rice, and maize (Dong, Chen & Chen, 2003; Ross, Liu & Shen, 2007; Hu et al., 2021). The *WRKY* family represents one of the largest groups of transcriptional regulators in plants (Mangelsen et al., 2008), characterized by the highly conserved WRKY domain. Each WRKY protein contains one or two conserved domains of approximately 60 amino acids, featuring a highly conserved WRKYGQK heptapeptide at the N-terminus and, in some cases, a zinc finger structure at the C-terminus, either C-X4–5-C-X22–23-HXH (C2H2) or C-X7-C-X23-HXC (C2HC) (Rushton et al., 2010). The WRKY domain exhibits a strong affinity for the conserved W-box (C/TTGACT/C) binding site, which is also present in multiple defense-related genes, highlighting its critical biological functions (Ulker & Somssich, 2004; Yamasaki et al., 2012). As versatile regulators, *WRKY* genes are involved in nearly all aspects of plant life, from growth and development to tolerance of various abiotic and biotic stresses (Wang et al., 2023). Overexpression of the *PtoWRKY60* gene in poplar plants can significantly enhance their resistance to *Dothiorella gregaria* Sacc (Ye et al., 2014). However, this also leads to adverse phenotypes such as slowed growth, premature leaf senescence and shedding. Several studies have highlighted the role of *WRKY* genes in herbivore-induced plant defense. For instance, silencing the *SlWRKY70* gene in tomato (*Lycopersicon esculentum*) inhibited the *Mi-1* gene-mediated anti-aphid pathway, increasing the susceptibility of mutant plants to aphids (Atamian, Eulgem & Kaloshian, 2012). Overexpression of the *OsWRKY89* gene enhanced rice resistance to the white-backed planthopper (Wang et al., 2007). However, despite these advancements, research on the defense mechanisms of *PtWRKY* genes targeting herbivorous insects remains limited, revealing a significant gap in current knowledge.

*Populus trichocarpa* serves as a model species for woody plants, with its genome sequence widely utilized in genetic studies since its sequencing and publication in 2006 (Tuskan et al., 2006). It is a key model plant for research on stress resistance in *Populus* due to its rapid growth, well-defined genetic background, and ease of genetic transformation (Liu et al., 2022; Xu et al., 2021). Although the *WRKY* gene family in *P. trichocarpa* has been previously analyzed, with subgroup III gene expression patterns studied under cold, drought, salinity, and salicylic acid stresses (He et al., 2012), a comprehensive analysis of *PtWRKY* genes under insect stress remains unexplored. In this study, the most recent *P. trichocarpa* genome was employed to identify all *WRKY* genes in the species. Detailed analyses, including molecular characterization, phylogenetic relationships, gene structure, motif and promoter composition, chromosomal localization, and syntenic relationships, were conducted. Real-time quantitative polymerase chain reaction (RT-qPCR) was utilized to assess the expression profiles of *PtWRKY* genes under insect stress. This research enhances the functional characterization (physicochemical properties, domains, evolutionary relationships, regulatory elements, and expression patterns under insect stress conditions). of *PtWRKY* genes and identifies key candidate genes for improving poplar resistance to pest stress.

## Materials and Methods

### Plant and insect materials and stress treatment

Three 2-month-old *P. trichocarpa* seedlings with consistent growth were selected for the experiment. The plants were randomly assigned to either a control or a treatment group. For each group, three independent plants were established as biological replicates. From the same node position (the 3rd–5th nodes) of each plant, three healthy and uniformly growing leaves were selected. *Hyphantria cunea* were provided by the Chinese Academy of Forestry Sciences, were incubated in an artificial climate chamber (L:D = 14:10; temperature 25 ± 1 °C; humidity 70 ± 5%) until the second instar. Prior to treatment, *H. cunea* larvae were subjected to a starvation protocol, with nine larvae starved for 12 h and placed evenly on the leaves. *P. trichocarpa* samples were collected 12 h after treatment. To ensure independence across time points, each sampling event used entirely new plants. All experiments were performed in triplicate, and the collected samples were immediately frozen in liquid nitrogen and stored at −80 °C for total RNA extraction. The samples collected at 0 h post-treatment served as the control group.

### Identification of *PtWRKY* genes

Genomic sequences, coding sequences, and protein sequences of *P. trichocarpa* were retrieved from the National Center for Biotechnology Information (GCF_000002775.5) database (https://www.ncbi.nlm.nih.gov). The 71 WRKY protein sequences from *A. thaliana* were downloaded from the TAIR (GCF_000001735.4) website (https://www.arabidopsis.org) as reference sequences. Using the WRKY protein sequence from *A. thaliana* as a query, the BLAST program was employed to compare sequences in the *P. trichocarpa* genome. Duplicates were merged and removed to obtain the candidate *WRKY* TF protein sequences of *P. trichocarpa*. Further identification of *PtWRKY* genes was carried out using the NCBI CD-search tool (https://www.ncbi.nlm.nih.gov/Structure/bwrpsb/bwrpsb.cgi) for protein domain analysis, ultimately leading to the identification of all *WRKY* genes in *P. trichocarpa*. Basic protein properties, including amino acid length, molecular weight (MW), theoretical isoelectric point (pI), instability index, aliphatic index, and grand average of hydropathicity (GRAVY), were predicted using the ExPASy ProtParam tool (https://web.expasy.org/protparam/). The subcellular localization of PtWRKY proteins was predicted using Plant-mPLoc (http://www.csbio.sjtu.edu.cn/bioinf/plant-multi/) (Chou & Shen, 2010).

### Construction of phylogenetic tree

Phylogenetic trees were constructed using the neighbor joining (NJ) method in MEGA11.0 software with a bootstrap value set to 1,000 (Kumar, Stecher & Tamura, 2016). The evolutionary tree was visualized using the iTOL online tool (http://iTOL.embl.de) (Letunic & Bork, 2019).

### Visualization of gene structure and conserved motif of *WRKY*

Conservative motif analysis of the *P. trichocarpa WRKY* family protein sequences was conducted using the MEME tool (https://meme-suite.org/meme/tools/meme) (Tang et al., 2008), setting the number of motifs to eight. Based on the *P*. *trichocarpa* genome GFF3 annotation file, the gene structure of *PtWRKY* genes was analyzed and visualized using TBtools software.

### Collinearity and chromosomal mapping of *WRKY*

The DNA sequences of the entire *WRKY* gene family in *P. trichocarpa* were mapped across the genome. The distribution of these genes on chromosomes and scaffolds was analyzed using TBtools software. Genome data for *A. thaliana*, *Salix purpurea*, and *Oryza sativa* were downloaded from the Ensembl website, organized, and covariance analysis across species was performed and visualized using TBtools software (Chen et al., 2020).

### Predicted co-expression and interaction network

For protein interaction networks, homologous WRKY proteins in Arabidopsis were identified using STRING (https://string-db.org) with a threshold value >0.7. The homologous proteins of the identified interactive *P. trichocarpa* proteins were determined by reciprocal best BLASTP analysis. The network was then visualized and analyzed using Cytoscape version 3.7.0 (Shannon et al., 2003).

### Analysis of cis-regulatory elements

The promoter sequences, extending 2,000 bp upstream of the start codon, were extracted from the *P. trichocarpa* genome file using TBtools software. Cis-acting elements within the *PtWRKY* gene promoters were identified using the online tool Plant Care (http://bioinformatics.psb.ugent.be/webtools/plantcare/html) (Lescot et al., 2002).

### RNA isolation and RT-qPCR

Total RNA was extracted from *P. trichocarpa* leaf (the third to fifth healthy and fully expanded leaves) tissues using a plant RNA extraction kit (Tiangen, Beijing, China). The RNA was reverse-transcribed into cDNA using a reverse transcription kit (TaKaRa, Beijing, China). RT-qPCR primers for candidate genes were designed with Primer Premier (version 5.0) (Table 1). Gene expression levels were quantified using the CFX Opus 96 Real-Time PCR system (Bio-Rad, Singapore) and TB Green Premix EX Taq II FAST qPCR (TaKaRa, Beijing, China). The PCR conditions were: 95 °C for 30 s, followed by 40 cycles of 95 °C for 5 s, 60 °C for 30 s, with melting curve conditions at 65 °C for 5 s and 95 °C for 5 s. The poplar actin gene served as the internal reference. The relative expression levels of each gene were calculated using the 2^−∆∆Ct^ method. It includes three cases of biological replication and three cases of technical replicates.

**Table 1:** The primer sequence information for RT-PCR.

ID	Gene name	Forward primer sequence (F)	Reverse primer sequence (R)
XM_002297947.4	*PtWRKY2*	TGAAGATCCCACCGTATTTGAG	GTTGTGGAAGTTAAAGAGGGC
XM_002298817.4	*PtWRKY3*	TCATCCTAAACCACAACCCAG	TTGGTTAGGCAGAGATGAAGAC
XM_002303816.4	*PtWRKY16*	AGTGGAATTGAACCGAGTGAG	CTCTCAGAGTGCCCATTCAT
XM_002309030.4	*PtWRKY26*	TCGGGTTTGGAAGCAATAGG	AGGCGGAGAACGGTTGAAGACAC
XM_002309150.4	*PtWRKY27*	GATCAATTTGTCTTGGACCCG	GATTGGAAGGATGTGGTAAAGAATG
XM_002311293.4	*PtWRKY30*	CAGTTACCCCGAGAAAATCCTC	GTACGATGTCCCCAAGCAG
XM_006369831.3	*PtWRKY54*	GTTCCAGCCACCCCTAATTC	TCCTCTGGCTTTTCTGGTTG
XM_024583529.2	*PtWRKY76*	GATCCCTACACATTCGAGGTG	AATTCCAGCCATCTAGCGAG
XM_024585451.2	*PtWRKY80*	TCAAGTACAATCACAGAACCCC	TTCTTTCAACGAGACCTCCAC
XM_024601099.2	*PtWRKY101*	AGCGGATATAGTGGTGCAAC	TGTGGGCTGTTCTTGACTG
XM_024604661.2	*PtWRKY107*	GCTCTGAAACGACAAGGAAATG	TTTCCACTTGCCCATCCC
XM_024606501.2	*PtWRKY112*	CAGCTTCTATCTCTTCCTCTTCG	GTCATGAAAGCAAATCTCGGC
	Actin	AATACCCCATTGAGCACGG	ACTCACACCATCACCAGAATC

## Results

### Identification and physicochemical property analysis of *PtWRKY* genes

Conserved domains were identified using NCBI-CDD and Pfam, resulting in the identification of 139 *PtWRKY* genes. Based on their chromosomal arrangement, these genes were renamed *PtWRKY1* through *PtWRKY139* (Table 2). Physicochemical property analysis revealed that the length of WRKY proteins ranged from 157 amino acids (*PtWRKY18*) to 739 amino acids (*PtWRKY81*), with significant variation in the number of amino acids. The MW varied between 17,992.37 Da (*PtWRKY18*) and 79,601.83 Da (*PtWRKY81*). The pI ranged from 4.98 (*PtWRKY129*) to 9.86 (*PtWRKY25*). All *PtWRKY* genes were hydrophilic, with a mean hydrophilicity index ranging from −1.1 to −0.493. Predicted subcellular localization indicated that all 139 *PtWRKY* genes are located in the nucleus.

**Table 2:** Physical and chemical properties of *PtWRKKY*.

Name	Gene ID	Family	WRKY area	Number of amino acid	Molecular weight/Da	Theoretical pI	Subcellular location
*PtWRKY1*	rna-XM_002297587.4	Group II-d	WRKYGQK	314	35,189.87	9.45	Nucleus
*PtWRKY2*	rna-XM_002297947.4	Group III	WRKYGQK	338	37966.72	5.79	Nucleus
*PtWRKY3*	rna-XM_002298817.4	Group I	WRKYGQK	557	60,421.64	6.53	Nucleus
*PtWRKY4*	rna-XM_002300084.4	Group II-c	WRKYGQK	186	21,227.88	9.44	Nucleus
*PtWRKY5*	rna-XM_002300560.4	Group I	WRKYGQK	731	78,739.83	6.16	Nucleus
*PtWRKY6*	rna-XM_002301130.4	Group II-d	WRKYGQK	358	39,612.68	9.51	Nucleus
*PtWRKY7*	rna-XM_002301201.4	Group II-c	WRKYGQK	203	23,424.71	7.64	Nucleus
*PtWRKY8*	rna-XM_002301341.4	Group II-c	WRKYGQK	193	21,894.03	9.06	Nucleus
*PtWRKY9*	rna-XM_002301478.4	Group II-d	WRKYGQK	245	27,692.54	5.71	Nucleus
*PtWRKY10*	rna-XM_002301488.4	Group II-c	WRKYGQK	317	35,402.53	6.67	Nucleus
*PtWRKY11*	rna-XM_002302034.4	Group II-d	WRKYGQK	351	38,916.09	9.74	Nucleus
*PtWRKY12*	rna-XM_002302104.4	Group II-c	WRKYGQK	325	36,596.37	7.65	Nucleus
*PtWRKY13*	rna-XM_002302584.4	Group III	WRKYGQK	363	40,939.24	5.82	Nucleus
*PtWRKY14*	rna-XM_002302772.4	Group II-b	WRKYGQK	506	54,489.39	7.56	Nucleus
*PtWRKY15*	rna-XM_002303574.4	Group II-e	WRKYGQK	330	37,033.59	5.49	Nucleus
*PtWRKY16*	rna-XM_002303816.4	Group II-a	WRKYGQK	319	35,674.03	8.49	Nucleus
*PtWRKY17*	rna-XM_002304513.4	Group III	WRKYGQK	342	38,391.23	5.25	Nucleus
*PtWRKY18*	rna-XM_002304705.4	Group II-c	WRKYGQK	157	17,992.37	9.58	Nucleus
*PtWRKY19*	rna-XM_002305235.4	Group II-e	WRKYGQK	432	47,090.98	5.08	Nucleus
*PtWRKY20*	rna-XM_002305694.4	Group II-d	WRKYGQK	262	29,293.51	5.56	Nucleus
*PtWRKY21*	rna-XM_002306707.4	Group II-c	WRKYGQK	322	36,311.07	7.19	Nucleus
*PtWRKY22*	rna-XM_002306787.4	Group II-d	WRKYGQK	347	38,556.76	9.64	Nucleus
*PtWRKY23*	rna-XM_002308502.3	Group II-c	WRKYGQK	165	18,800.72	5.48	Nucleus
*PtWRKY24*	rna-XM_002308668.3	Group II-a	WRKYGQK	320	35,415.62	8.27	Nucleus
*PtWRKY25*	rna-XM_002308962.4	Group II-d	WRKYGQK	301	32,898.03	9.86	Nucleus
*PtWRKY26*	rna-XM_002309030.4	Group II-a	WRKYGQK	304	34,029.43	5.52	Nucleus
*PtWRKY27*	rna-XM_002309150.4	Group III	WRKYGQK	333	37,705.54	6.04	Nucleus
*PtWRKY28*	rna-XM_002310008.4	Group II-c	WRKYGKK	233	26,378.91	9.32	Nucleus
*PtWRKY29*	rna-XM_002310086.4	Group II-d	WRKYGQK	335	36,803.7	9.65	Nucleus
*PtWRKY30*	rna-XM_002311293.4	Group II-c	WRKYGQK	293	32,254.6	5.85	Nucleus
*PtWRKY31*	rna-XM_002312231.4	Group I	WRKYGQK	492	54,144	8.7	Nucleus
*PtWRKY32*	rna-XM_002312282.4	Group II-c	WRKYGQK	368	40,893.57	7.06	Nucleus
*PtWRKY33*	rna-XM_002314926.4	Group II-c	WRKYGQK	374	41,381.84	6.2	Nucleus
*PtWRKY34*	rna-XM_002314988.4	Group I	WRKYGQK	499	54,536.98	8.57	Nucleus
*PtWRKY35*	rna-XM_002317361.4	Group II-d	WRKYGQK	268	30,051.11	5.78	Nucleus
*PtWRKY36*	rna-XM_002318337.4	Group III	WRKYGQK	371	41,419.78	5.56	Nucleus
*PtWRKY37*	rna-XM_002318711.4	Group II-c	WRKYGQK	186	21,494.98	8.9	Nucleus
*PtWRKY38*	rna-XM_002319046.4	Group II-d	WRKYGQK	354	40,094.08	9.79	Nucleus
*PtWRKY39*	rna-XM_002319843.4	Group III	WRKYGQK	324	36,719.84	5.38	Nucleus
*PtWRKY40*	rna-XM_002319923.4	Group I	WRKYGQK	591	64,943.87	7.2	Nucleus
*PtWRKY41*	rna-XM_002320124.4	Group II-c	WRKYGQK	189	21,456.47	9.22	Nucleus
*PtWRKY42*	rna-XM_002320816.4	Group III	WRKYGQK	365	41,209.55	5.1	Nucleus
*PtWRKY43*	rna-XM_002320931.4	Group II-c	WRKYGQK	319	35,894.11	7.06	Nucleus
*PtWRKY44*	rna-XM_002322238.4	Group II-c	WRKYGQK	178	20,390.76	9.42	Nucleus
*PtWRKY45*	rna-XM_002323601.4	Group I	WRKYGQK	579	63,971.87	6.2	Nucleus
*PtWRKY46*	rna-XM_002323803.4	Group II-b	WRKYGQK	538	58,374.23	6.26	Nucleus
*PtWRKY47*	rna-XM_002324292.4	Group II-a	WRKYGQK	271	30,475.4	7.59	Nucleus
*PtWRKY48*	rna-XM_002324346.4	Group II-d	WRKYGQK	338	36,823.47	9.45	Nucleus
*PtWRKY49*	rna-XM_002325212.4	Group II-d	WRKYGQK	300	33,025.26	9.81	Nucleus
*PtWRKY50*	rna-XM_002326057.4	Group I	WRKYGQK	603	66,358.41	6.69	Nucleus
*PtWRKY51*	rna-XM_006368449.3	Group II-a	WRKYGQK	318	35,131.43	8.85	Nucleus
*PtWRKY52*	rna-XM_006368706.3	Group II-e	WRKYGQK	325	36,713.96	5.69	Nucleus
*PtWRKY53*	rna-XM_006368806.3	Group II-c	WRKYGQK	160	18,342.73	9.58	Nucleus
*PtWRKY54*	rna-XM_006369831.3	Group II-c	WRKYGQK	312	34,893.44	6.31	Nucleus
*PtWRKY55*	rna-XM_006371700.3	Group II-a	WRKYGQK	320	35,569.97	9.03	Nucleus
*PtWRKY56*	rna-XM_006372308.3	Group II-e	WRKYGQK	412	45,622.66	5.65	Nucleus
*PtWRKY57*	rna-XM_006373154.3	Group II-c	WRKYGQK	186	21,250.89	9.61	Nucleus
*PtWRKY58*	rna-XM_006375106.3	Group II-c	WRKYGQK	228	26,409.89	6.26	Nucleus
*PtWRKY59*	rna-XM_006375289.3	Group II-e	WRKYGQK	349	38,367.88	6.16	Nucleus
*PtWRKY60*	rna-XM_006375493.3	Group I	WRKYGQK	485	53,397.61	5.98	Nucleus
*PtWRKY61*	rna-XM_006375494.3	Group I	WRKYGQK	485	53,397.61	5.98	Nucleus
*PtWRKY62*	rna-XM_006377952.3	Group I	WRKYGQK	725	78,446.32	5.83	Nucleus
*PtWRKY63*	rna-XM_006380631.3	Group II-d	WRKYGQK	334	36,716.62	9.65	Nucleus
*PtWRKY64*	rna-XM_006381457.3	Group I	WRKYGQK	475	51,973.49	9.12	Nucleus
*PtWRKY65*	rna-XM_006382750.3	Group II-d	WRKYGQK	353	39,696.51	9.69	Nucleus
*PtWRKY66*	rna-XM_006383468.3	Group II-d	WRKYGQK	331	36,663.4	9.38	Nucleus
*PtWRKY67*	rna-XM_006386554.3	Group II-e	WRKYGQK	354	38,846.56	6.15	Nucleus
*PtWRKY68*	rna-XM_006389516.2	Group II-b	WRKYGQK	590	64,185.08	6.61	Nucleus
*PtWRKY69*	rna-XM_024581109.2	Group II-c	WRKYGQK	306	34,030.86	8.16	Nucleus
*PtWRKY70*	rna-XM_024581374.2	Group I	WRKYGQK	725	78,446.32	5.83	Nucleus
*PtWRKY71*	rna-XM_024581375.2	Group I	WRKYGQK	725	78,446.32	5.83	Nucleus
*PtWRKY72*	rna-XM_024581376.2	Group I	WRKYGQK	708	76,572.32	5.92	Nucleus
*PtWRKY73*	rna-XM_024581601.2	Group I	WRKYGQK	560	60,801.21	6.97	Nucleus
*PtWRKY74*	rna-XM_024581602.2	Group I	WRKYGQK	507	55,043.94	6.94	Nucleus
*PtWRKY75*	rna-XM_024583061.2	Group I	WRKYGQK	716	78,369.07	6.62	Nucleus
*PtWRKY76*	rna-XM_024583529.2	Group III	WRKYGQK	364	40,549.27	6.09	Nucleus
*PtWRKY77*	rna-XM_024584301.2	Group II-b	WRKYGQK	502	54,065.41	6.9	Nucleus
*PtWRKY78*	rna-XM_024584519.2	Group I	WRKYGQK	485	53,397.61	5.98	Nucleus
*PtWRKY79*	rna-XM_024585445.2	Group I	WRKYGQK	739	79,601.83	6.16	Nucleus
*PtWRKY80*	rna-XM_024585451.2	Group I	WRKYGQK	739	79,601.83	6.16	Nucleus
*PtWRKY81*	rna-XM_024585457.2	Group I	WRKYGQK	739	79,601.83	6.16	Nucleus
*PtWRKY82*	rna-XM_024585466.2	Group I	WRKYGQK	732	78,906	6.23	Nucleus
*PtWRKY83*	rna-XM_024585549.2	Group II-d	WRKYGQK	361	39,868.07	9.36	Nucleus
*PtWRKY84*	rna-XM_024585550.2	Group II-d	WRKYGQK	360	39,780.99	9.36	Nucleus
*PtWRKY85*	rna-XM_024587515.2	Group I	WRKYGQK	469	51,535.26	9.34	Nucleus
*PtWRKY86*	rna-XM_024587516.2	Group I	WRKYGQK	469	51,535.26	9.34	Nucleus
*PtWRKY87*	rna-XM_024587806.2	Group I	WRKYGQK	556	60,364.59	6.53	Nucleus
*PtWRKY88*	rna-XM_024588647.2	Group I	WRKYGQK	532	58,247.79	7.75	Nucleus
*PtWRKY89*	rna-XM_024590285.2	Group I	WRKYGQK	527	57,897.46	5.69	Nucleus
*PtWRKY90*	rna-XM_024590287.2	Group I	WRKYGQK	523	57,483.05	5.82	Nucleus
*PtWRKY91*	rna-XM_024590516.2	Group II-c	WRKYGQK	511	56,542.75	9.13	Nucleus
*PtWRKY92*	rna-XM_024590518.2	Group II-c	WRKYGQK	422	46,295.02	9.26	Nucleus
*PtWRKY93*	rna-XM_024594046.2	Group II-d	WRKYGQK	357	39,525.6	9.51	Nucleus
*PtWRKY94*	rna-XM_024594979.2	Group II-c	WRKYGQK	261	29,161.58	9.18	Nucleus
*PtWRKY95*	rna-XM_024596020.2	Group II-d	WRKYGQK	245	27,692.54	5.71	Nucleus
*PtWRKY96*	rna-XM_024596958.2	Group II-d	WRKYGQK	304	34,405.91	9.47	Nucleus
*PtWRKY97*	rna-XM_024598510.2	Group I	WRKYGQK	543	59,495.25	7.27	Nucleus
*PtWRKY98*	rna-XM_024598865.2	Group II-d	WRKYGQK	223	25,044.07	6.08	Nucleus
*PtWRKY99*	rna-XM_024600078.2	Group II-b	WRKYGQK	602	65,078.08	6.29	Nucleus
*PtWRKY100*	rna-XM_024600511.2	Group II-d	WRKYGQK	332	36,750.48	9.38	Nucleus
*PtWRKY101*	rna-XM_024601099.2	Group II-c	WRKYGKK	206	22,873.15	6.06	Nucleus
*PtWRKY102*	rna-XM_024602206.2	Group II-d	WRKYGQK	347	38,556.76	9.64	Nucleus
*PtWRKY103*	rna-XM_024602795.2	Group II-e	WRKYGQK	448	48,448.32	5.63	Nucleus
*PtWRKY104*	rna-XM_024602932.2	Group I	WRKYGQK	546	60,333.25	8.9	Nucleus
*PtWRKY105*	rna-XM_024603986.2	Group I	WRKYGQK	475	51,973.49	9.12	Nucleus
*PtWRKY106*	rna-XM_024603990.2	Group I	WRKYGQK	475	51,973.49	9.12	Nucleus
*PtWRKY107*	rna-XM_024604661.2	Group I	WRKYGQK	522	56,833.44	6.32	Nucleus
*PtWRKY108*	rna-XM_024604662.2	Group I	WRKYGQK	518	56,419.02	6.57	Nucleus
*PtWRKY109*	rna-XM_024605234.2	Group II-c	WRKYGKK	192	21,887.15	7.63	Nucleus
*PtWRKY110*	rna-XM_024606155.2	Group II-c	WRKYGQK	293	32,254.6	5.85	Nucleus
*PtWRKY111*	rna-XM_024606156.2	Group II-c	WRKYGQK	293	32,254.6	5.85	Nucleus
*PtWRKY112*	rna-XM_024606501.2	Group II-c	WRKYGQK	318	35,270.42	7.01	Nucleus
*PtWRKY113*	rna-XM_024609281.2	Group II-d	WRKYGQK	313	35,033.68	9.39	Nucleus
*PtWRKY114*	rna-XM_024610899.2	Group II-b	WRKYGQK	523	57,025.65	6.09	Nucleus
*PtWRKY115*	rna-XM_024611133.2	Group II-c	WRKYGQK	293	32,595.97	6.4	Nucleus
*PtWRKY116*	rna-XM_024611531.2	Group II-b	WRKYGQK	593	64,574.49	6.61	Nucleus
*PtWRKY117*	rna-XM_024611532.2	Group II-b	WRKYGQK	592	64,446.32	6.48	Nucleus
*PtWRKY118*	rna-XM_024611533.1	Group II-b	WRKYGQK	589	64,056.91	6.48	Nucleus
*PtWRKY119*	rna-XM_052445140.1	Group II-e	WRKYGQK	461	49,731.65	5.71	Nucleus
*PtWRKY120*	rna-XM_052445501.1	Group I	WRKYGQK	541	59,246.01	7.95	Nucleus
*PtWRKY121*	rna-XM_052445816.1	Group II-c	WRKYGQK	185	21,338.79	8.68	Nucleus
*PtWRKY122*	rna-XM_052446276.1	Group III	WRKYGQK	324	36,719.84	5.38	Nucleus
*PtWRKY123*	rna-XM_052447277.1	Group II-d	WRKYGQK	262	29,899.11	5.26	Nucleus
*PtWRKY124*	rna-XM_052447278.1	Group II-d	WRKYGQK	235	26,838.72	5.07	Nucleus
*PtWRKY125*	rna-XM_052447365.1	Group II-b	WRKYGQK	636	68,314.18	6.49	Nucleus
*PtWRKY126*	rna-XM_052449326.1	Group III	WRKYGQK	381	41,276.09	6.1	Nucleus
*PtWRKY127*	rna-XM_052450880.1	Group II-d	WRKYGQK	245	27,917.09	5.96	Nucleus
*PtWRKY128*	rna-XM_052450881.1	Group II-d	WRKYGQK	245	27,917.09	5.96	Nucleus
*PtWRKY129*	rna-XM_052450883.1	Group II-d		209	23,823.39	4.98	Nucleus
*PtWRKY130*	rna-XM_052452687.1	Group II-c	WRKYGQK	232	26,308.64	9.08	Nucleus
*PtWRKY131*	rna-XM_052452688.1	Group II-c	WRKYGQK	232	26,308.64	9.08	Nucleus
*PtWRKY132*	rna-XM_052453559.1	Group I	WRKYGQK	475	51,973.49	9.12	Nucleus
*PtWRKY133*	rna-XM_052453560.1	Group I	WRKYGQK	475	51,973.49	9.12	Nucleus
*PtWRKY134*	rna-XM_052453561.1	Group I	WRKYGQK	475	51,973.49	9.12	Nucleus
*PtWRKY135*	rna-XM_052453562.1	Group I	WRKYGQK	475	51,973.49	9.12	Nucleus
*PtWRKY136*	rna-XM_052454988.1	Group I	WRKYGQK	731	78,739.83	6.16	Nucleus
*PtWRKY137*	rna-XM_052455199.1	Group II-c	WRKYGQK	293	32,254.6	5.85	Nucleus
*PtWRKY138*	rna-XM_052455200.1	Group II-c	WRKYGQK	293	32,254.6	5.85	Nucleus
*PtWRKY139*	rna-XM_052456799.1	Group II-c	WRKYGQK	293	32,595.97	6.4	Nucleus

### Phylogenetic analysis and multiple sequence alignment of *PtWRKY* genes

**Figure 1:**
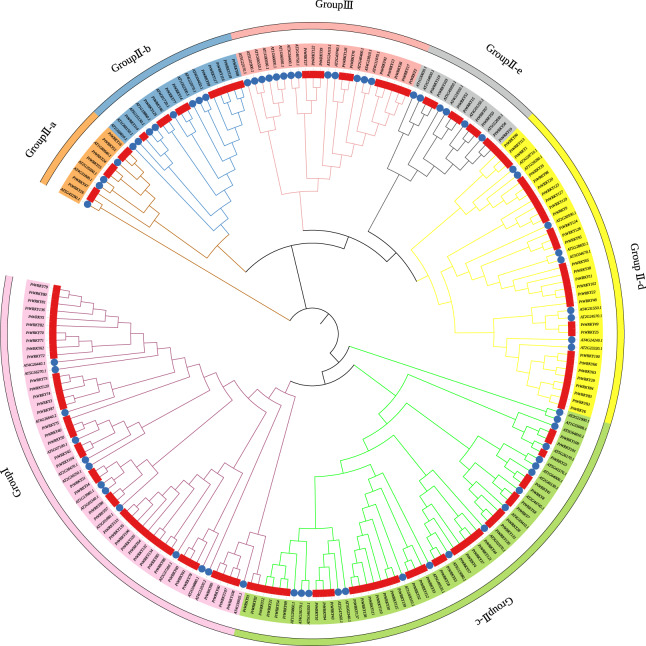
Phylogenetic tree of WRKY proteins.

Phylogenetic analysis was conducted using the 139 WRKY protein sequences from *P. trichocarpa* and 71 known WRKY protein sequences from *A. thaliana*. The analysis revealed that the 139 *P. trichocarpa WRKY* TFs could be classified into three major groups (Fig. 1). Group I contained 40 WRKY proteins, group II contained 88 WRKY proteins, and group III contained 10 WRKY proteins. Group II was further subdivided into five subclasses: IIa (six members), IIb (10 members), IIc (36 members), IId (28 members), and IIe (eight members). Based on evolutionary relationships, the *WRKY* family in higher plants can be divided into three subclasses: IIa + IIb, IIc, and IId + IIe, consistent with the phylogenetic tree results. Multiple sequence alignment (Table 2) showed that 135 of the 139 PtWRKY proteins contained the signature WRKYGQK conserved domain. Four proteins deviated: *PtWRKY129* (subgroup IIc) lacked the WRKY domain, while *PtWRKY28*, *PtWRKY101*, and *PtWRKY109* exhibited mutations in the conserved domain, replacing WRKYGQK with WRKYGKK.

### Conserved motif and exon–intron structure analyses of *PtWRKY* genes

**Figure 2:**
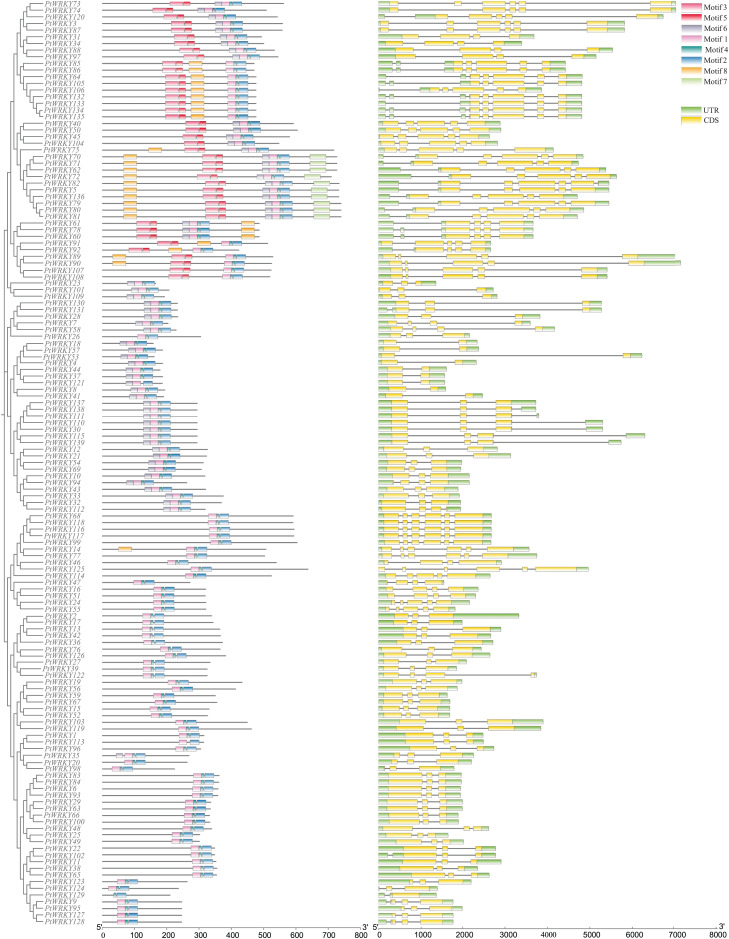
PtWRKY conserved motifs and gene structure.

To investigate the structural diversity of the *PtWRKY* genes, a rootless evolutionary tree was constructed to analyze conserved motifs and gene structures (Fig. 2). Gene structure analysis revealed that all *PtWRKY* genes contain introns, with the number of exons ranging from two (*PtWRKY26*, *PtWRKY18*, *PtWRKY53*, *PtWRKY57*, *PtWRKY4*, *PtWRKY44*, *PtWRKY8*, *PtWRKY37*, *PtWRKY121*, *PtWRKY41*) to six (*PtWRKY73*, *PtWRKY3*, *PtWRKY87*, *PtWRKY68*, *PtWRKY119*, *PtWRKY118*, *PtWRKY117*, *PtWRKY99*, *PtWRKY14*, *PtWRKY77*, *PtWRKY125*). The number of introns varied between one and five in the *PtWRKY* genes. Notably, most *PtWRKY* genes within the same group exhibited similar exon-intron structures (Figs. 2B and 2C).

**Figure 3:**
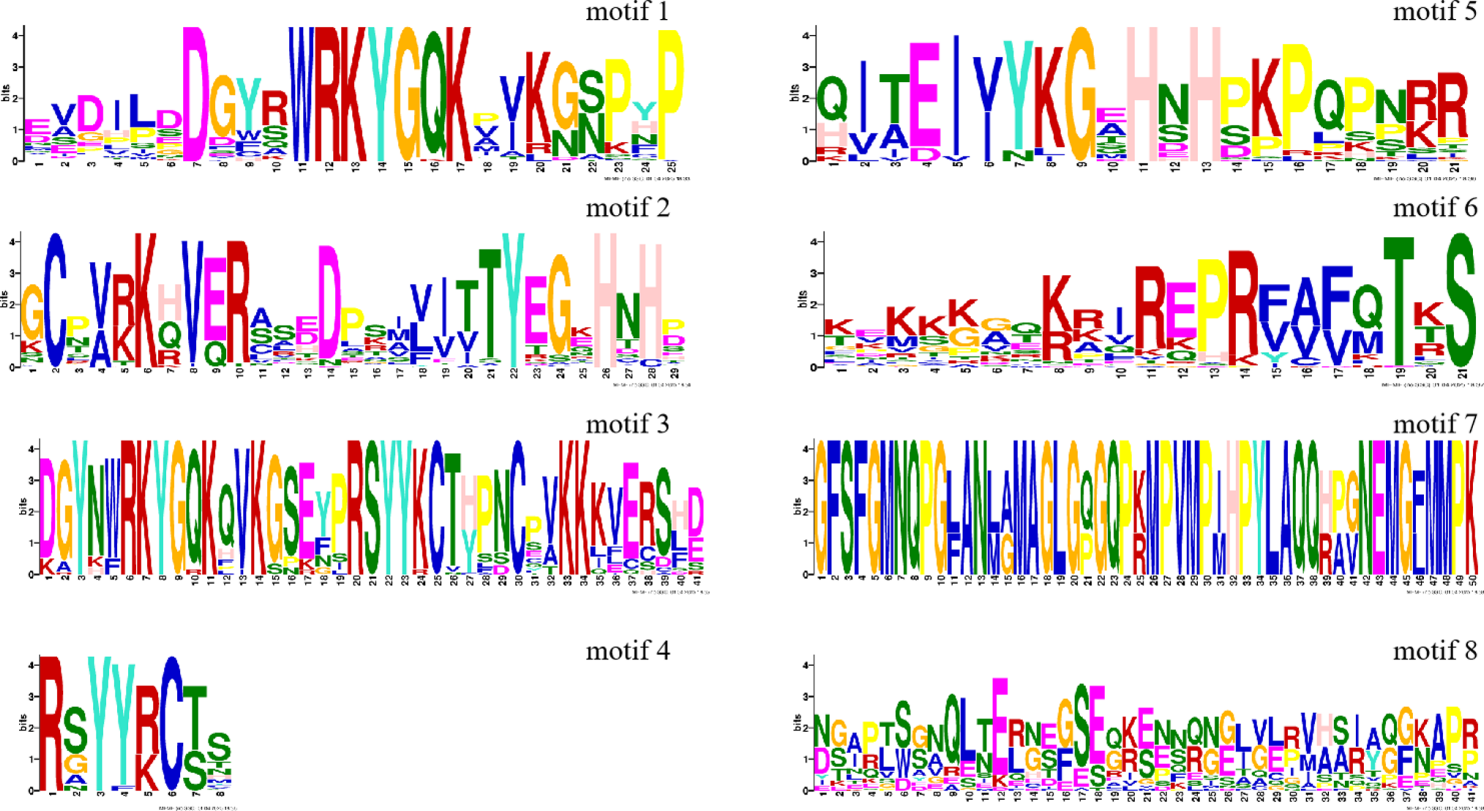
Conserved motifs of the PtWRKY protein.

To further explore the structural features and conduct optimal functional analyses of PtWRKY proteins, conserved motifs in the 139 PtWRKY proteins were examined using the MEME online software. This analysis identified eight highly correlated conserved motifs (Fig. 3). The frequency with which each motif occurred within WRKY proteins indicates its significance in the sequence. Notably, motifs 1 and 3 correspond to the conserved WRKYGQK heptapeptide segment. All *PtWRKY* proteins in the family possess motifs 1, 2, and 4, which represent the core conserved domains of the *PtWRKY* genes. These motifs play a critical role in maintaining the fundamental characteristics and functional integrity of the gene family.

### Collinearity analysis of *WRKY* gene family members in *P. trichocarpa*

**Figure 4:**
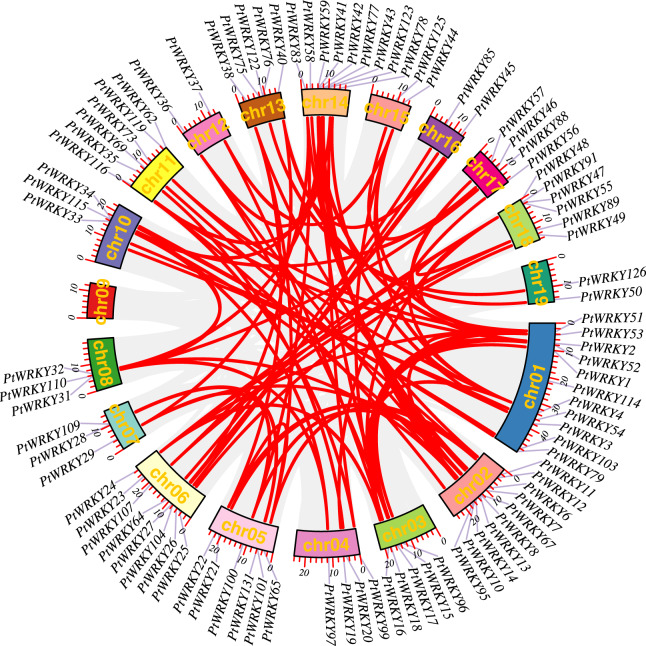
Distribution of WRKY genes.

During evolution, segmental duplication, whole-genome duplication (WGD), and tandem duplications have played key roles in gene family expansion in plants. In the case of *PtWRKY* genes, intra-species collinearity analysis revealed fourteen pairs of tandem duplication events, primarily located on chromosomes Chr01, Chr02, Chr06, Chr11, Chr13, Chr14, and Chr18. Evolutionary analysis of the 139 *PtWRKY* genes identified 91 genes originating from WGD events (Fig. 4), suggesting that WGD was the major contributor to the expansion of the *WRKY* gene family in *Populus*.

**Figure 5:**
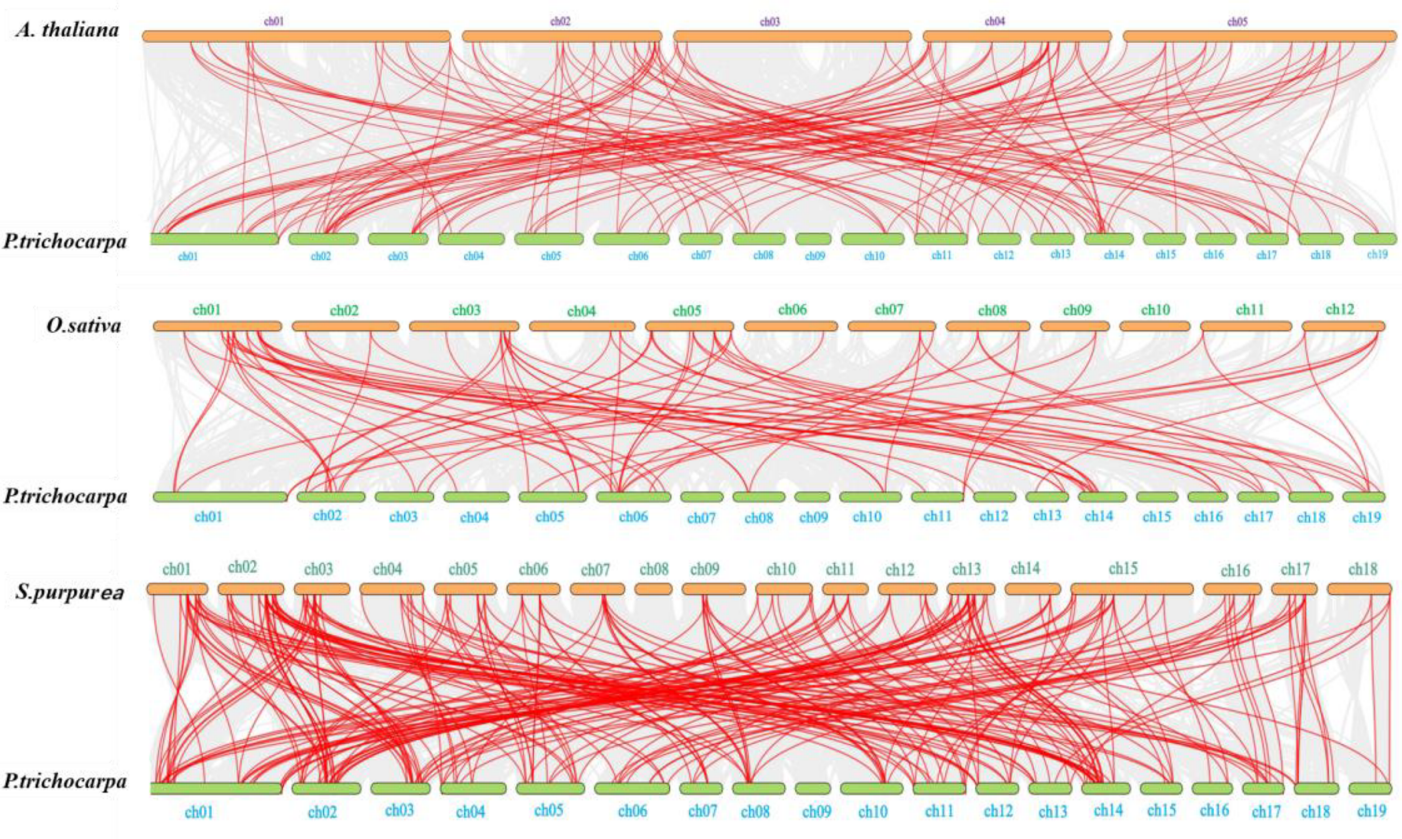
Synteny analysis of WRKY genes between *P. trichocarpa* and three representative plant species.

To further investigate the phylogenetic relationship of *WRKY* genes between *P. trichocarpa* and three other species, interspecific collinearity analysis was performed (Fig. 5). The numbers of orthologous *WRKY* pairs between *P. trichocarpa* and *S. purpurea*, *A. thaliana*, and *O. sativa* were 235, 123, and 75, respectively. These results indicate stronger collinearity between *P. trichocarpa* and *S. purpurea*, followed by *A. thaliana*, and finally *O. sativa*.

### Chromosome mapping of *PtWRKY* genes

**Figure 6:**
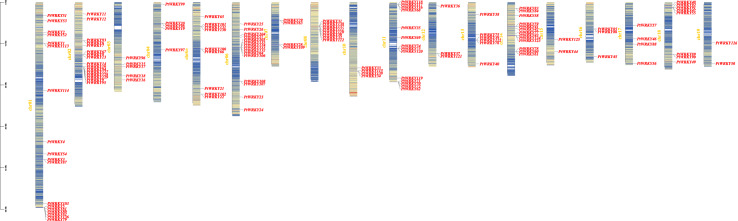
Chromosomal location of the WRKY gene family in *P. trichocarpa*.

Using TBtools software, the *PtWRKY* genes were mapped to *P. trichocarpa* chromosomes (Fig. 6). The results revealed that these genes are distributed across 18 chromosomes (excluding Chr09), though the distribution and density across chromosomes are uneven. Specifically, the highest number of genes (18) is located on Chr01, while the lowest (only two genes) is found on Chr19. These results confirm the uneven distribution and density of *PtWRKY* genes across the chromosomes, despite their presence on 18 chromosomes.

### Prediction of co-regulatory and interaction networks of *PtWRKYs*

**Figure 7:**
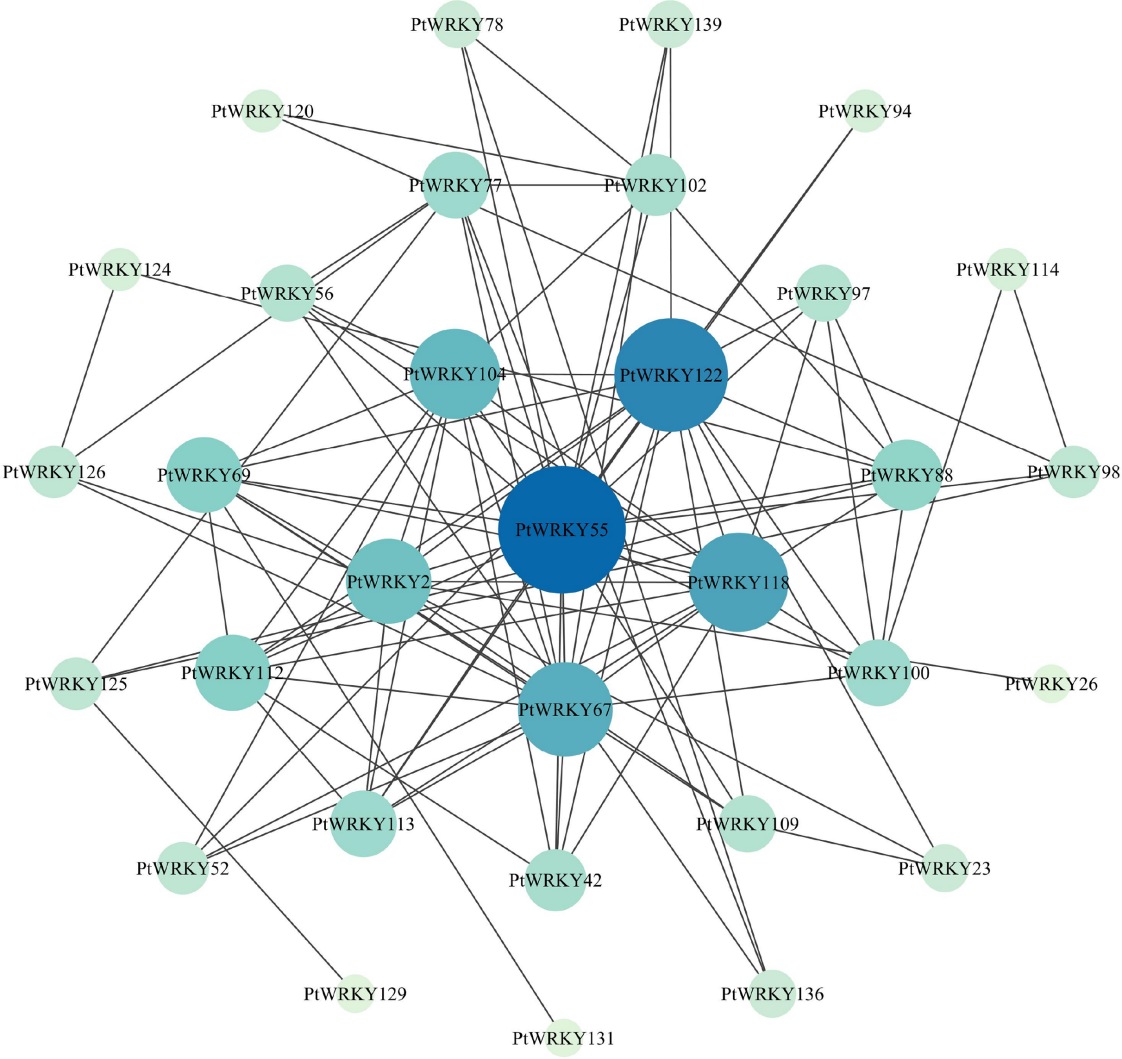
Protein-protein interaction (PPI) network of important genes.

*WRKY* TFs represent one of the largest families of transcriptional regulators in plants, integral to signaling networks that modulate various plant processes. To explore the potential regulatory networks among *PtWRKY* genes, co-expression patterns for the 139 PtWRKY proteins were predicted using the STRING protein-protein interaction database, revealing that 32 genes are involved in interactions (Fig. 7). Notably, *PtWRKY55* and *PtWRKY122* occupy central positions in the network, displaying extensive connections with numerous other PtWRKY proteins. In contrast, peripheral nodes such as *PtWRKY94*, *PtWRKY131*, and *PtWRKY129* exhibit relatively fewer connections. These results suggest that *PtWRKY55* and *PtWRKY122* likely play pivotal roles in regulating the PtWRKY protein network, potentially acting as hub regulators that mediate interactions among a wide range of *WRKY* family members to coordinate various plant biological processes.

### Cis-acting elements analysis of *WRKY* gene family members in *P. trichocarpa*

**Figure 8:**
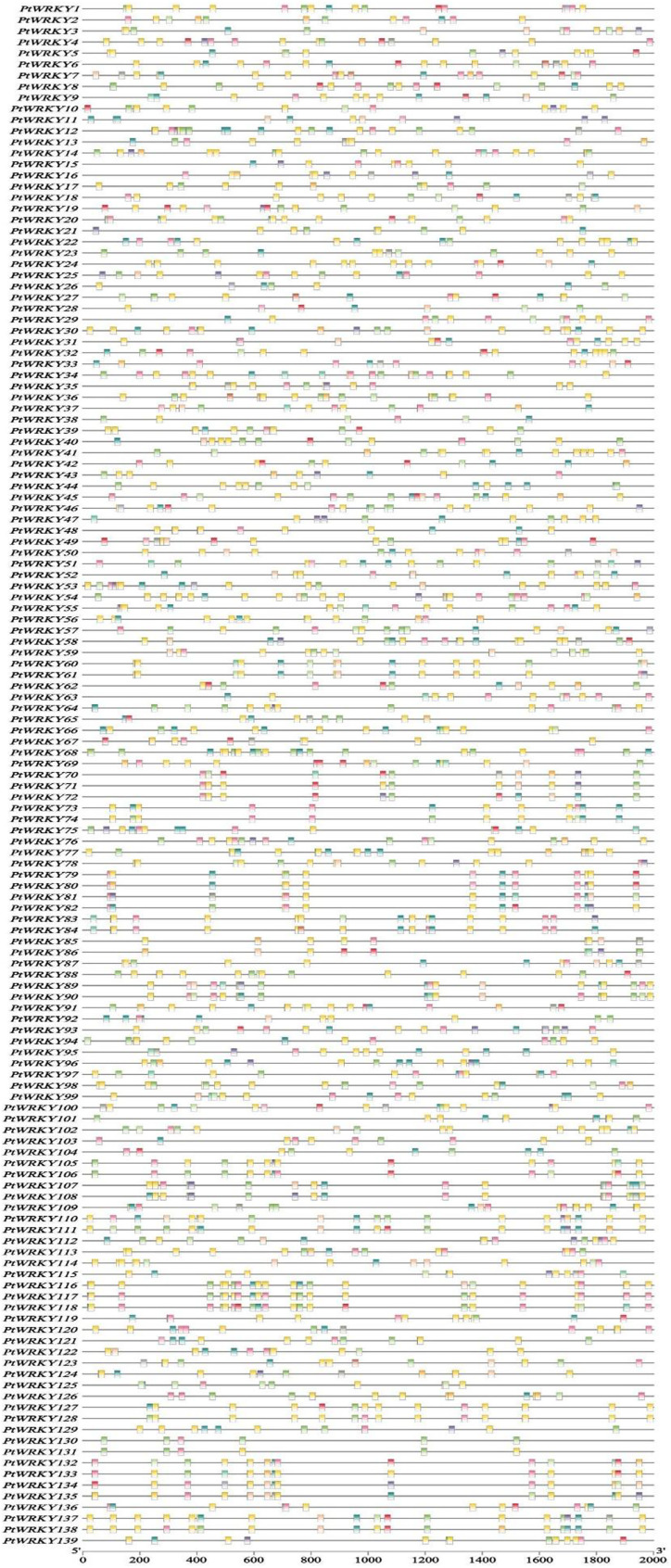
Cis-acting elements of the Pt WRKY gene family.

To further investigate the transcriptional regulation and potential functions of *PtWRKY* genes, PlantCARE was used to predict the *cis*-acting elements in their promoters. The analysis revealed that, in addition to promoter-related elements and *WRKY* binding site motifs, three types of *cis*-regulatory elements were highly concentrated in the *PtWRKY* gene promoters: light-responsive elements, plant hormone response elements, and environmental stress response-related elements (Fig. 8). Among these, the environmental stress-related elements included low-temperature (LTR), drought (MBS), defense and stress (Tc-rich repeats), and anaerobic induction (ARE) response elements. Light-responsive elements, such as Box4 and G-box, were the most abundant, with all 139 *PtWRKY* genes containing these motifs. Plant hormone response elements included methyl jasmonate-responsive elements (CGTCA-Motif and TGACG-Motif), abscisic acid-responsive elements (ABRE), auxin responsiveness (AuxRR-core), salicylic acid-responsive elements (TCA-element), and gibberellin-responsive elements (p-box and GARE-motif). These results suggest that the majority of cis-acting elements in *PtWRKY* genes are associated with stress responses. Analyzing these elements can provide valuable insights into how *PtWRKY* genes mediate stress responses, particularly under biotic stress conditions.

### Expression analysis of *PtWRKY* genes under *H. cunea* feeding

**Figure 9:**
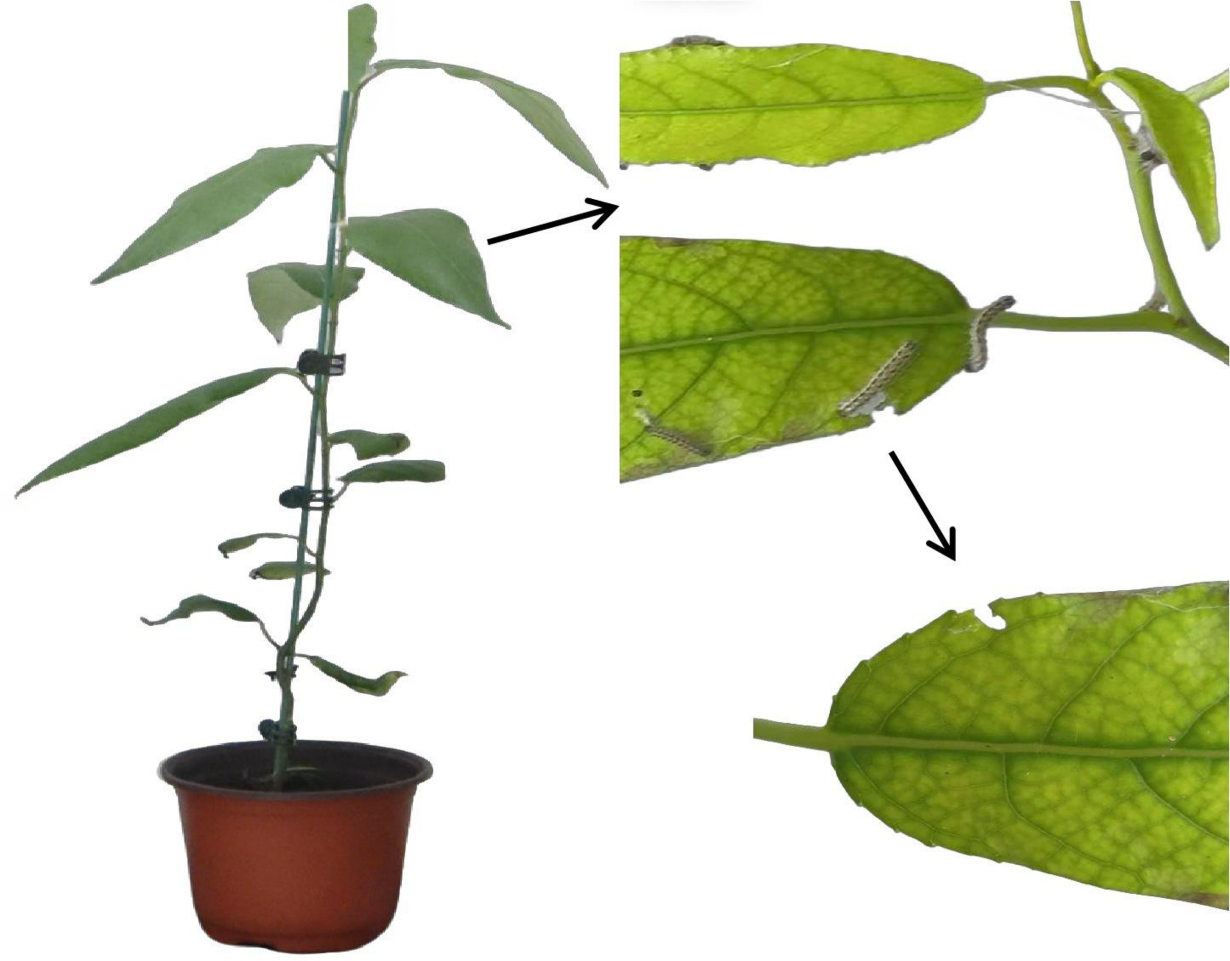
Plants of *Populus trichocar*, *Hyphantria cunea larvae* feeding *leaves*.

**Figure 10:**
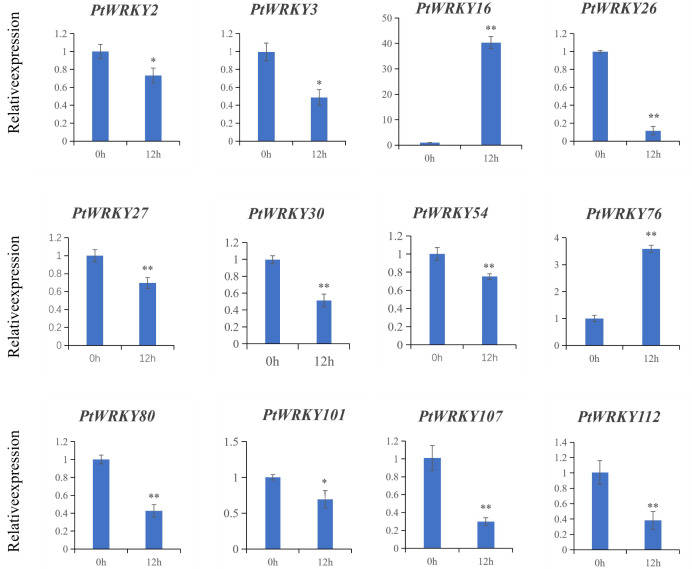
Relative expression levels of all WRKYs under *H.cunea* treatments.

Jasmonic acid (JA) and salicylic acid (SA) are key regulators of plant defense mechanisms against insect herbivores, while abscisic acid (ABA) also plays a role in responding to various stresses, including biotic stresses induced by insects. Based on the central roles of JA, SA, and ABA in plant defense against herbivorous insects, we analyzed the 2,000-bp promoter sequences of the 139 *PtWRKY* genes using PlantCARE. We specifically prioritized genes containing *cis*-acting elements responsive to all three hormones, as these genes are likely regulated by multi-hormonal signals and involved in the defense of *P. trichocarpa* against *H. cunea*. (Fig. 9). To explore the role of the *WRKY* gene family in plant defense against *H. cunea*, the expression of 12 *PtWRKY* genes was analyzed using RT-qPCR (Fig. 10). Differences between the *H. cunea* feeding treatment and the control group were assessed using Test statistics (*t*-values) (* 0.01< *p* < 0.05; **p < 0.01). Except for *PtWRKY2, PtWRK3* and *PtWRKY101*, which exhibited significant differences, all the other genes showed extremely significant differences (Table 3). The results showed varied expression patterns, which may be attributed to their distinct regulatory functions or the complex interactions of TFs. After *H. cunea* feeding, the expression levels of *PtWRKY2* and *PtWRKY3* did not change significantly. In contrast, *PtWRKY16* and *PtWRKY76* were upregulated in response to insect feeding compared to the control (Fig. 10). The expression of other genes, including, *PtWRKY26*, *PtWRKY80*, *PtWRKY107*, and *PtWRKY112*, was significantly downregulated following insect feeding.

**Table 3:** Test statistics.

Gene	*T*-value	*p*-value
*PtWRKY2*	3.826	0.019
*PtWRKY3*	4.011	0.016
*PtWRKY16*	6.61	0.003
*PtWRKY26*	30.575	0.000
*PtWRKY27*	5.846	0.004
*PtWRKY30*	9.420	0.002
*PtWRKY54*	5.597	0.005
*PtWRKY76*	−25.022	0.000
*PtWRKY80*	11.753	0.000
*PtWRKY101*	3.933	0.017
*PtWRKY107*	8.417	0.001
*PtWRKY112*	5.623	0.005

## Discussion

*WRKY* TFs are critical regulators, forming one of the largest families of transcriptional regulators with broad involvement in biotic stress responses. *P. trichocarpa* possesses a notably higher number of *WRKY* family members compared to several other plant species, including *A. thaliana* (Dong, Chen & Chen, 2003), *G. hirsutum* (Dou et al., 2014), *Nelumbo nucifera* (Li et al., 2019), *Prunus persica* (Chen et al., 2016), and *Ananas comosus* (Xie et al., 2018). This variation is likely attributed to *P. trichocarpa*’s large genome size and the high frequency of repeat events. Using genome data, 139 *PtWRKY* genes were identified and subjected to comprehensive bioinformatics analysis. Evolutionary analysis classified the *PtWRKY* genes into three primary groups, with group II further divided into five subgroups (Fig. 1), consistent with classifications observed in other plant species (Huang et al., 2012; Liu et al., 2017; Yang et al., 2015).

Structural analysis revealed that the amino acid sequences of most *PtWRKY* genes contained the conserved heptapeptide sequence (WRKYGQK), although a few exhibited heptapeptide variants (WRKYGKK, WRAYGGK, and WKKHGEK). These mutations could potentially result in functional changes. In this study, the conserved motif WRKYGQK in the *PtWRKY28*, *PtWRKY101*, and *PtWRKY109* proteins, belonging to group IIc, was altered to WRKYGKK. Notably, the tobacco (*Nicotiana tabacum*) *NtWRKY12*, which contains a WRKYGKK motif, recognizes a downstream binding sequence (TTTTCCAC) that deviates significantly from the W-box (van Verk et al., 2008). Similarly, *GmWRKY6* and *GmWRKY21* in soybean (Zhou et al., 2008), also containing the WRKYGKK motif, exhibit abnormal binding to the W-box. It is hypothesized that these *PtWRKY* heptapeptide variants of *P. trichocarpa* may impair the WRKY protein’s ability to bind to DNA, or potentially enable recognition of novel motifs, leading to the emergence of new functions.

The creation of introns is a significant event in genomic evolution, contributing to species adaptation over time (Zhou et al., 2008; Bong-Seok & Choi, 2015; Patthy, 1999). Gene structure plays a pivotal role in the evolution of multigene families (Penny et al., 2009; Gaba et al., 2023). The number of introns influences plant responses to different developmental stages and environmental stimuli (Ohta, 2013; Liu et al., 2023). In this study, gene structure analysis of *PtWRKY* genes was integrated with phylogenetic evolution results. Evolutionary studies often classify proteins based on gene structure and motif arrangement relationships (Wiegmann et al., 2019; Hwarari et al., 2023). In this context, motifs 1, 2, and 4, which contain the *WRKY* domain, are identified as essential components of PtWRKY proteins. Additionally, PtWRKY proteins were categorized based on gene structure and motif configurations. The *PtWRKY* genes varied in exon count, ranging from two to six exons, and the exon-intron arrangements were consistent within each subfamily. The conserved structural motifs in PtWRKY proteins within the same subgroup were largely identical, suggesting that these proteins are relatively conserved during evolution. However, differences in the conserved motifs between subgroups indicate functional differentiation of PtWRKY proteins in each group.

A significant number of *PtWRKY* genes were located in duplicated genomic regions, highlighting the importance of large-scale duplication events in the expansion of the *PtWRKY* gene family. Ninety-one collinearity *PtWRKY* gene pairs were identified in the *P. trichocarpa* genome, emphasizing the role of segmental duplication in the expansion of these genes. Evolutionary analysis also revealed substantial synteny between *P. trichocarpa*, *A. thaliana*, *O. sativa*, and *S. purpurea*, indicating a shared ancestry prior to the divergence of these lineages. Interspecies collinearity analysis further showed that more *WRKY* gene pairs are present in *P. trichocarpa* and *S. purpurea*, suggesting closer genetic similarities between these two tree species. Consequently, the *PtWRKY* gene family is more closely related to *S. purpurea*, followed by *A. thaliana*, while *O. sativa* is the most distantly related. These findings collectively suggest that the *WRKY* gene family has undergone varying degrees of expansion across different plant species, aiding their adaptation to diverse environments.

In response to stress, plants adapt to adverse environments through signal transduction and molecular regulation mechanisms. TFs play a critical role in signal transduction by activating or inhibiting the transcription of downstream genes, specifically binding to gene promoter regions, thus modulating the expression of related functional genes (Schwechheimer, Zourelidou & Bevan, 1998). WRKY proteins bind to W-box elements ((C/T)TGAC(T/C)) or other cis-acting elements in the promoter regions of target genes, subsequently regulating the expression of downstream genes (Sun et al., 2003). Analysis of the *PtWRKY* gene promoters revealed several cis-acting elements responsive to plant hormones and various stresses, including W-box (*WRKY* TF-binding element), ABRE, ARE (auxin response element), SARE, and MeJARE. These findings suggest that *PtWRKY* genes may encode key regulators in *P. trichocarpa*’s stress response mechanisms, mediated through plant hormone pathways.

WRKY proteins are essential in mitigating stress-induced damage and enhancing stress tolerance by activating defense-related genes *via* signal transduction pathways. These pathways involve key hormones such as ABA (Lim et al., 2022; Liu et al., 2015; Rushton et al., 2012), JA (Hou et al., 2019; Huang et al., 2022; Nguyen, Goossens & Lacchini, 2022), and SA (Cui et al., 2020; van Verk, Bol & Linthorst, 2011). To investigate whether *PtWRKY* genes are involved in *P. trichocarpa* defense against the insect herbivore *H. cunea via* these hormonal pathways, we analyzed the promoter regions of all 139 *PtWRKY* genes sing PlantCARE. The analysis revealed that a significant subset harbors cis-acting elements responsive to JA, SA, and ABA, providing basics that their expression may be modulated by these defense-related signals. In this study, the analysis of the promoter region of the *PtWRKY* genes revealed that a part of genes contained cis-acting elements responsive to JA, SA, and ABA. This is consistent with the established role of JA in anti-herbivore defense—where it can be upregulated by WRKY proteins, JA is upregulated by *WRKY3* and *WRKY6* in Nattemata during insect attacks (Skibbe et al., 2008). *BpWRKY6* expression was induced by JA treatment, and the JA content of *BpWRKY6*-overexpressing birch was also greater than that of the control. Furthermore, *Y1H*, *EMSA*, *LUC*, and *ChIP*–qPCR results verified that *BpWRKY6* can bind to the promoters of JA synthesis genes to promote their expression (Xie et al., 2025). With the documented interplay between SA (Lortzing et al., 2019), ABA (Dinh, Baldwin & Galis, 2013), and JA (Bruce & Pickett, 2007) in enhancing plant resistance.

In this study, the expression levels of 12 *PtWRKY* genes were analyzed following 12 h of exposure to *H. cunea* feeding. Notably, *PtWRKY16*and *PtWRKY76* were upregulated in response to insect feeding, compared to the control. Other *WRKY* genes exhibited low expression after 12 h of exposure to *H. cunea*. This divergence in expression profiles occurred despite all candidate genes containing cis-elements for JA, SA, and/or ABA, suggesting they are subject to distinct hormonal regulation—where specific signals may dominate or be suppressed—during the early defense response. Several key candidate *PtWRKY* genes were identified in this study, which could have practical applications in enhancing insect resistance in *P. trichocarpa* in the field. Further experimentation involving transgenic plants that either silence or overexpress these candidate *WRKY* genes is necessary to directly identify the transcriptional targets of key *WRKY* genes. This will provide deeper insights into their roles in *P. trichocarpa*’s defense responses to *H. cunea*.

## Conclusions

This study identified a total of 139 *WRKY* gene family members in *P. trichocarpa*. These genes were classified into five subgroups based on phylogenetic relationships and primarily contained conserved Motifs 1, 2, and 4. The cis-acting elements in the promoters of these *WRKY* genes were associated with hormone signaling (JA, SA, ABA, *etc*.), light response, and defense and stress responses. Collinearity analysis revealed a stronger *WRKY* gene collinearity between *P. trichocarpa* and *S. purpurea*, highlighting conserved *WRKY* evolution within the Salicaceae family. Following *H. cunea* feeding, *PtWRKY16* and *PtWRKY76* were upregulated, indicating their role in biotic stress. In summary, this study provides valuable insights into the functional characteristics of *PtWRKY* genes and contributes to the identification of key stress response genes involved in *P. trichocarpa*’s defense against *H. cunea* in this field.

## Supplemental Information

10.7717/peerj.21132/supp-1 Supplemental Information one RT-PCR Data.

10.7717/peerj.21132/supp-2 Supplemental Information 2 MIQE checklist.

## Additional Information and Declarations

### Data Availability

The following information was supplied regarding data availability:

The data is available in the Supplemental File and at NCBI: GCF_000002775.5.
